# Supplementary material for: Phospho-islands and the evolution of phosphorylated amino acids in mammals
Source: PeerJ. 2020 Dec 2;8:e10436. doi: 10.7717/peerj.10436 (PMC7718798; doi:10.7717/peerj.10436)
Supplement: Supplemental Information 1 [file peerj-08-10436-s001.docx]

**Supplementary materials for “Phospho-islands and the evolution of phosphorylated amino acids in mammals”**

1. **Supplementary Figures**


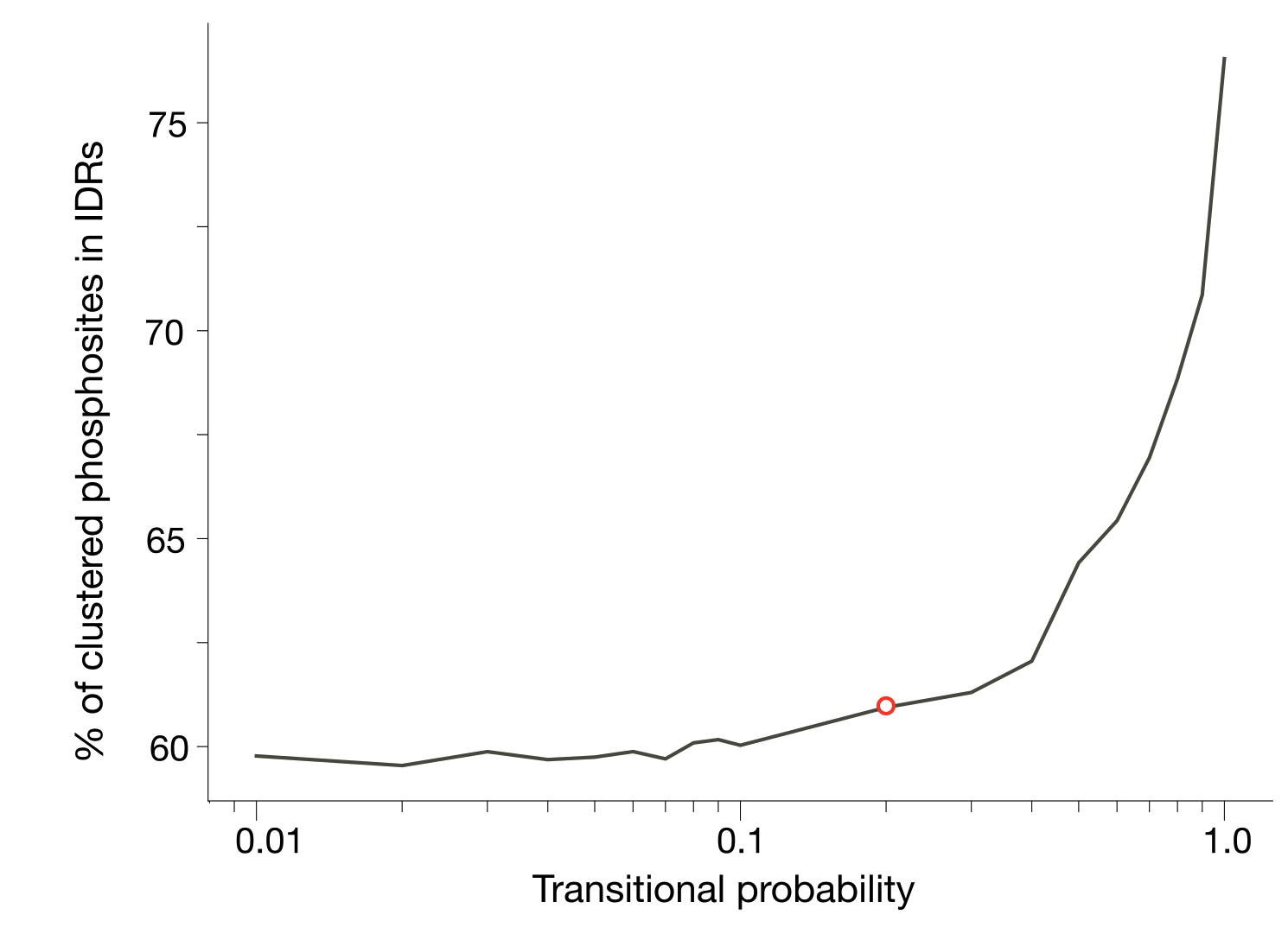


**Supplementary Figure S1 | Stability of hidden Markov model clustered editing site predictions with respect to transitional probability values.** Red circle represents the chosen value 0.2.


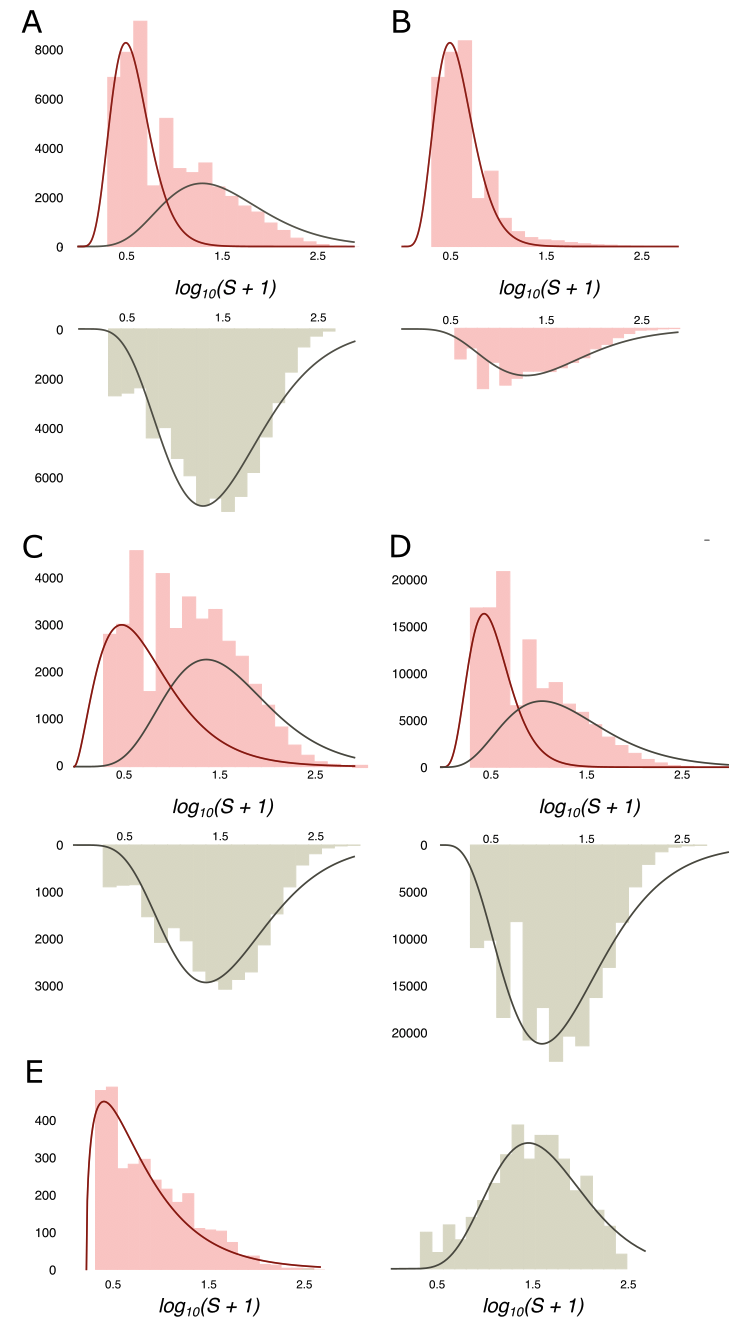


**Supplementary Figure S2 | Phospho-island analysis in various datasets.** **(A, B)** Prediction of phospho-islands for the mouse dataset. **(A)** (Above) The distribution of log_10_(*S* + 1) values (pink histogram; see the main text for definitions) and its decomposition in two gamma distributions, for phospho-islands (red curve) and for individual phosphosites (red curve). (Below) log_10_(*S +* 1) values for non-phosphorylated STY amino acids randomly sampled from IDRs with the same sample size and amino acid content as in the HMR dataset. **(B)** (Above) The distribution of log_10_(*S* + 1) values for phosphosites predicted to be in phospho-islands. (Below) log_10_(*S* + 1) values for predicted individual phosphosites. **(C)** Histograms of log_10_(*S* + 1) values for real (pink) and randomly assigned (grey) phosphosites with respective gamma-approximations for the rarefied human dataset. **(D)** Histograms of *S* values for real (pink) and randomly assigned (grey) phosphosites with respective gamma-approximations for the human dataset. **(E)** Distributions of the log_10_(*S* + 1) values of phosphosites located in ORs (pink) and the distribution of log_10_(*S* + 1) for STY amino acids randomly sampled from ORs (grey) with respective gamma-approximation. Note the good fit of a single gamma-distribution for the log_10_(*S* + 1) values observed in ORs.


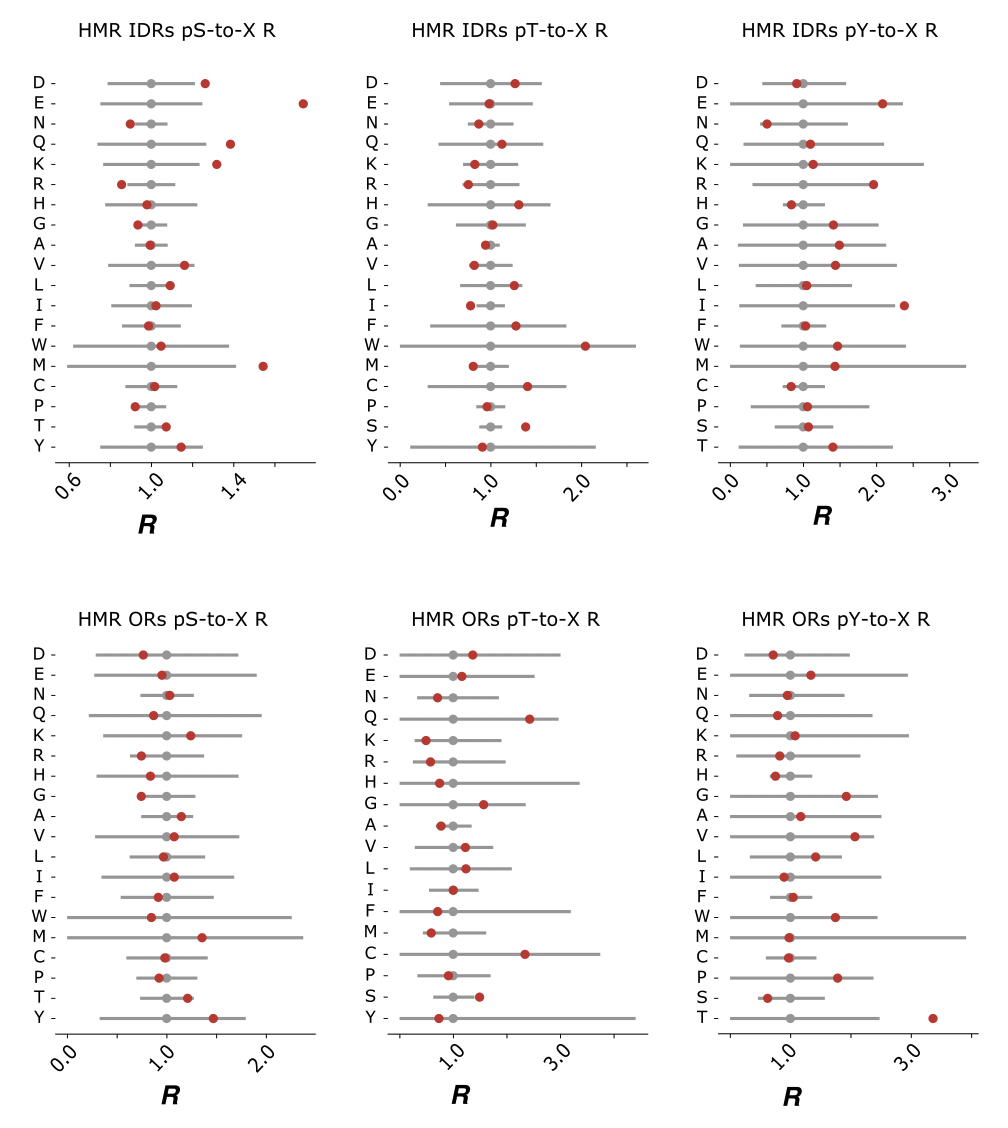


**Supplementary Figure S3 | Comparison of the mutational patterns observed in HMR phosphosites located in intrinsically disordered and ordered regions.** *R* values are defined in the main text. 95% two-tail confidence intervals are generated from the сhi-squared distribution with the Bonferroni correction. Red dots mark the actual *R* values. Grey dots represent the respective *R* values of non-phosphorylated STY amino acids.


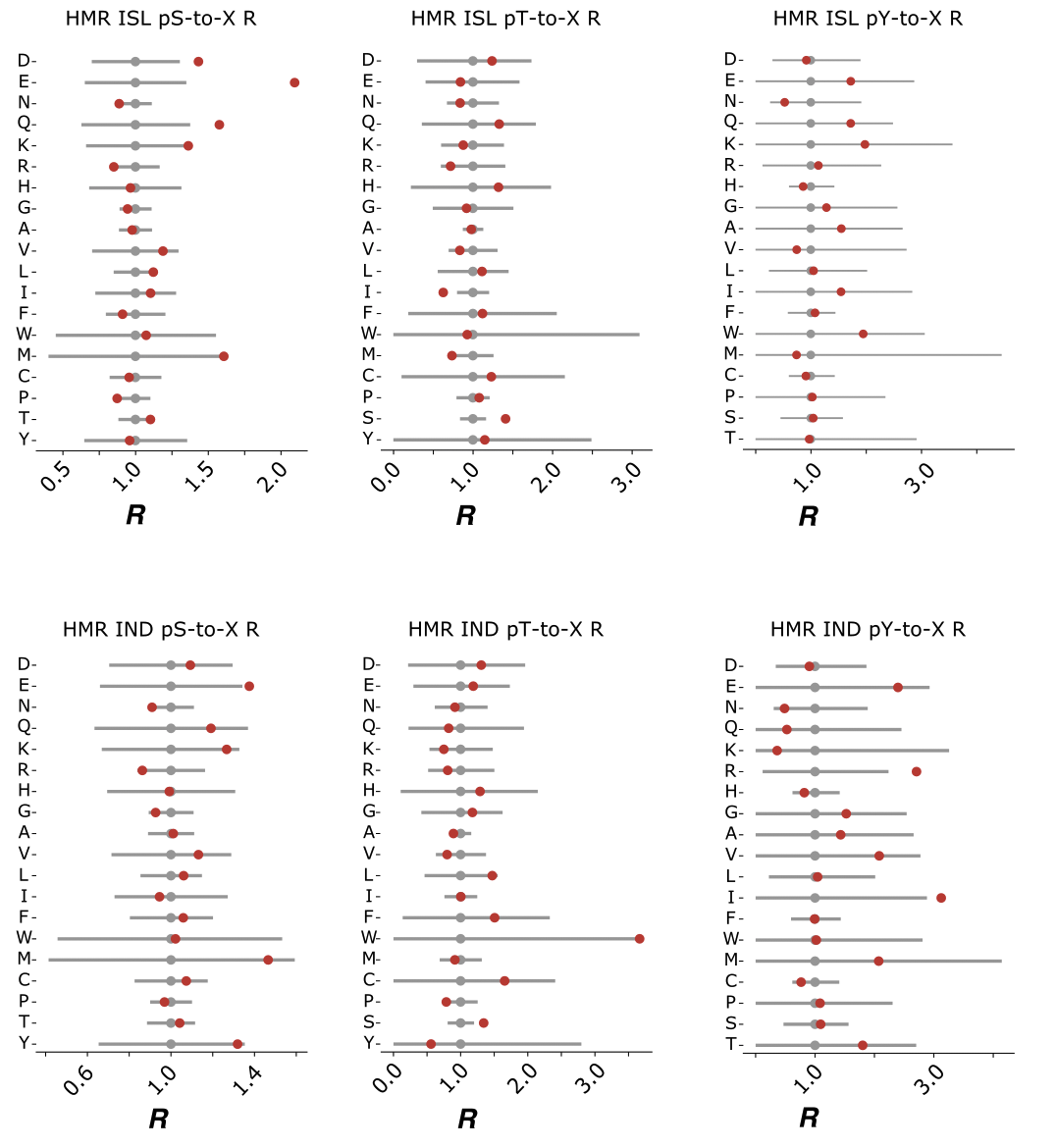


**Supplementary Figure S4 | Comparison of the mutational patterns observed in HMR phosphosites located in phospho-islands *vs*. individual ones.** Notation as in Suppl. Fig. S2.


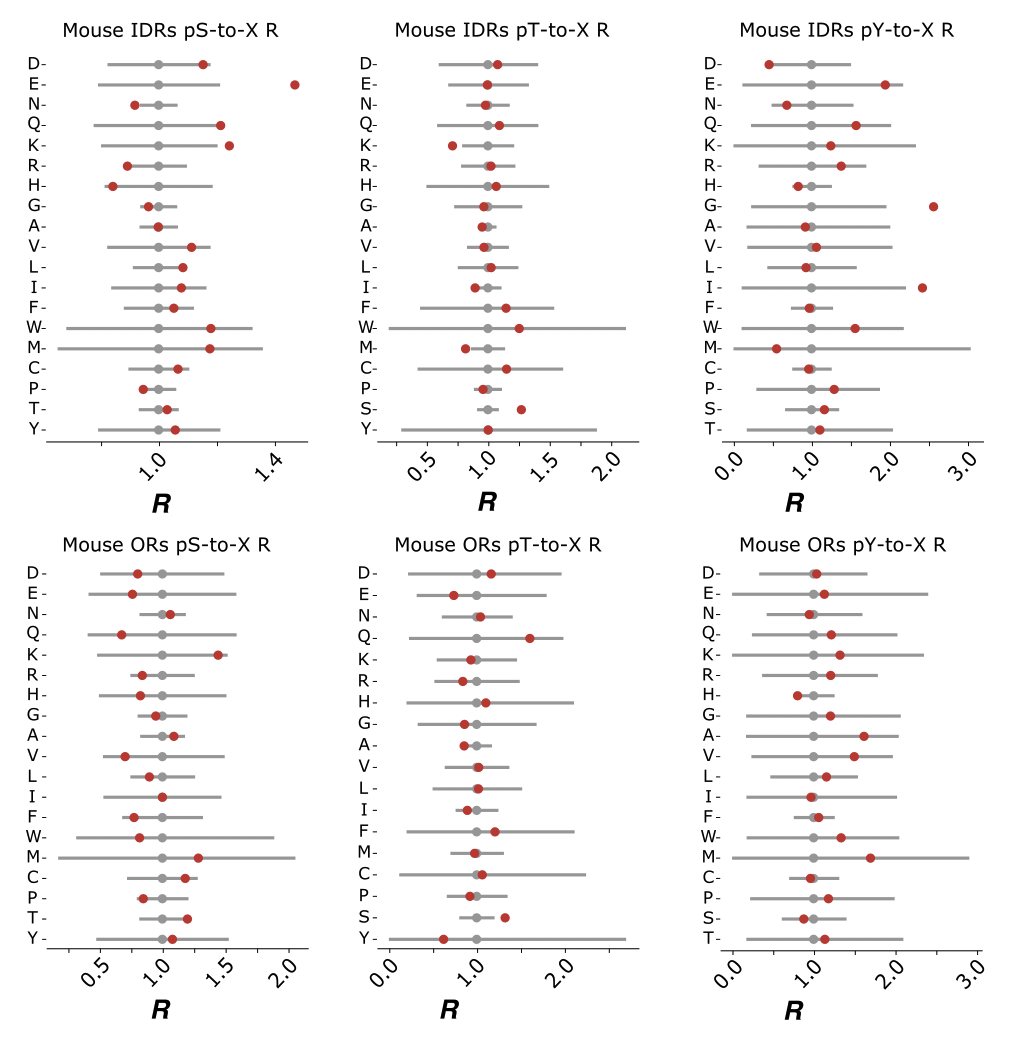


**Supplementary Figure S5 | Comparison of the mutational patterns observed in mouse phosphosites located in intrinsically disordered and ordered regions.** Notation as in Suppl. Fig. S2.


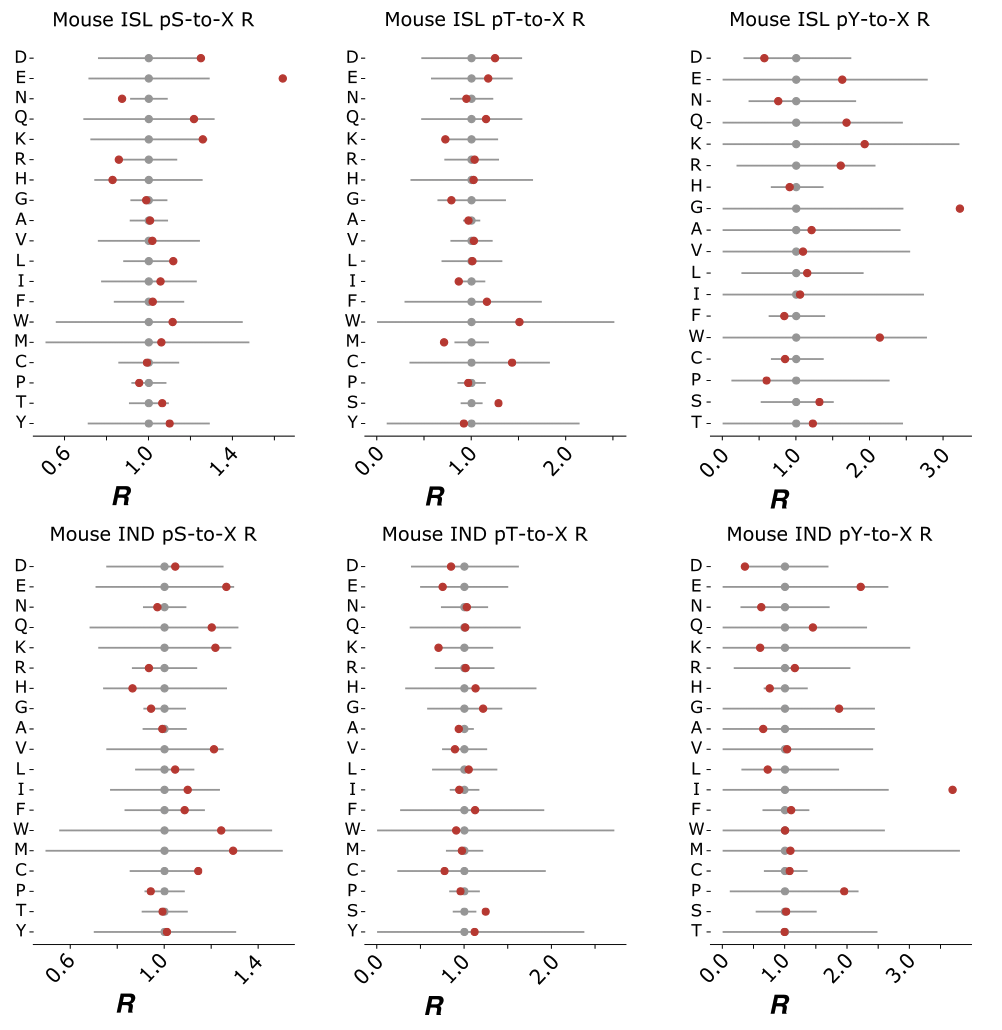


**Supplementary Figure S6 | Comparison of the mutational patterns observed in mouse phosphosites located in phospho-islands *vs*. individual ones.** Notation as in Suppl. Fig. S2.


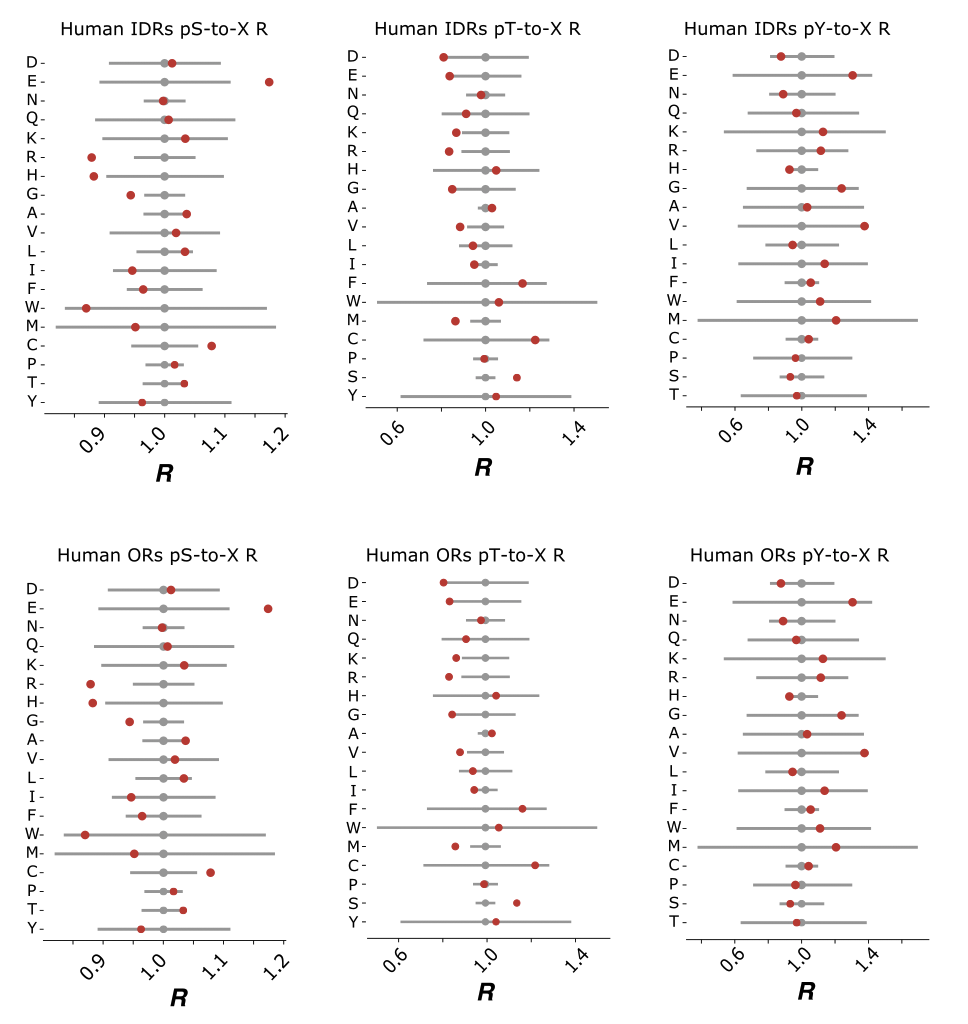


**Supplementary Figure S7 | Comparison of the mutational patterns observed in human phosphosites located in intrinsically disordered and ordered regions.** Notation as in Suppl. Fig. S2.


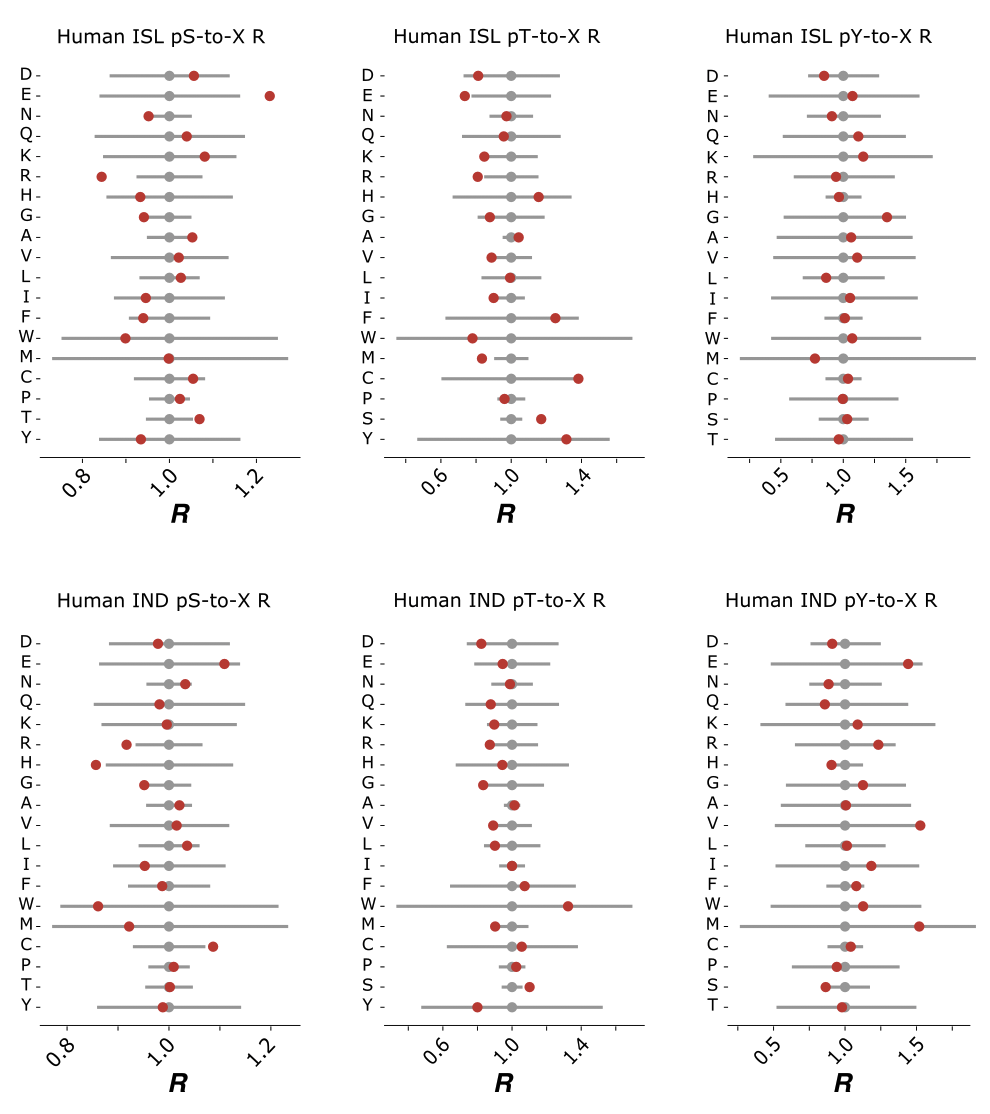


**Supplementary Figure S8 | Comparison of the mutational patterns observed in human phosphosites located in phospho-islands *vs*. individual ones.** Notation as in Suppl. Fig. S2.


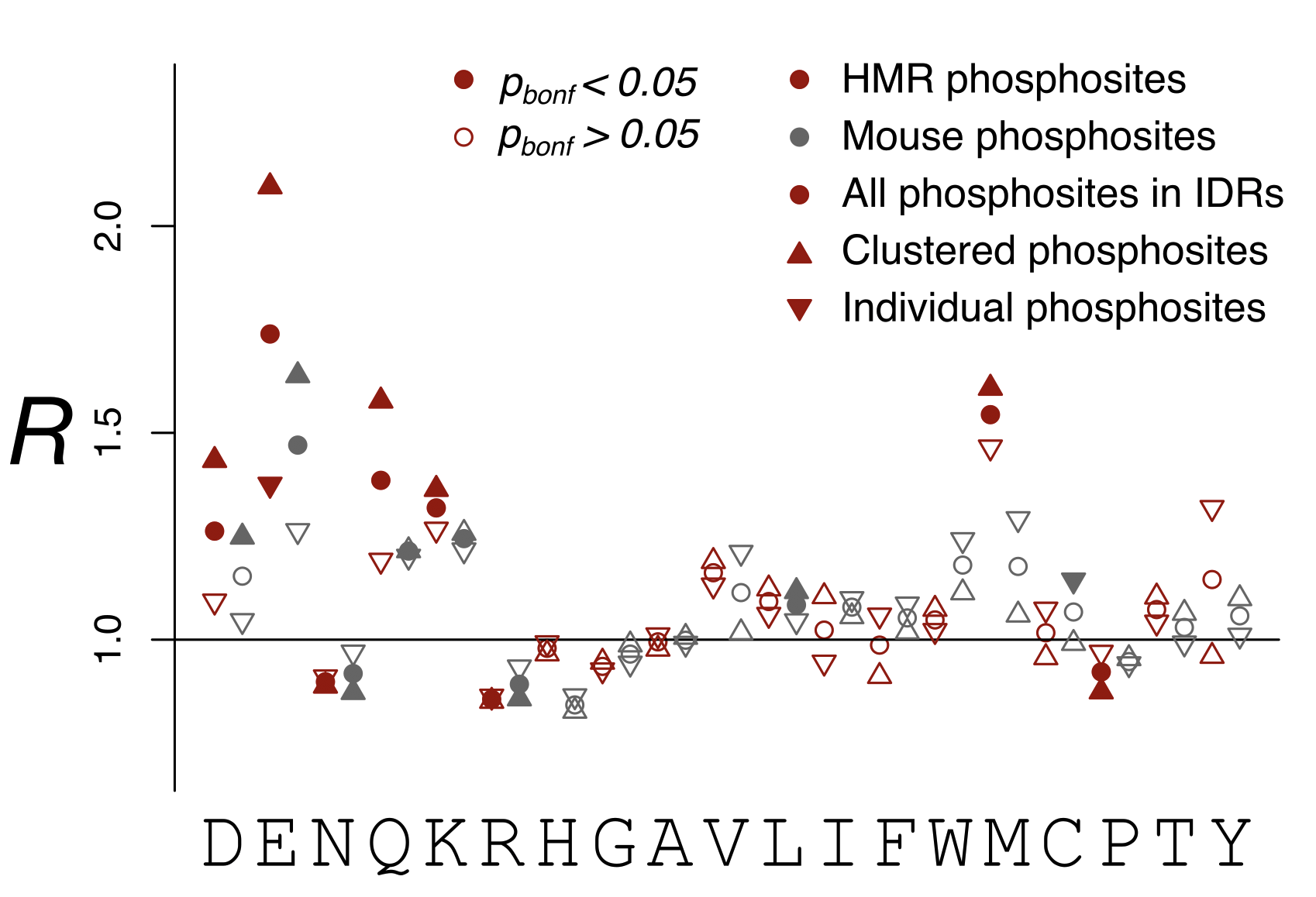


**Supplementary Figure S9 | *R* values of the pS-to-X mutations for different phosphosite sets.** Note the stronger effects observed for the HMR dataset compared to the mouse dataset and for clustered phosphoserines with respect to individual ones.


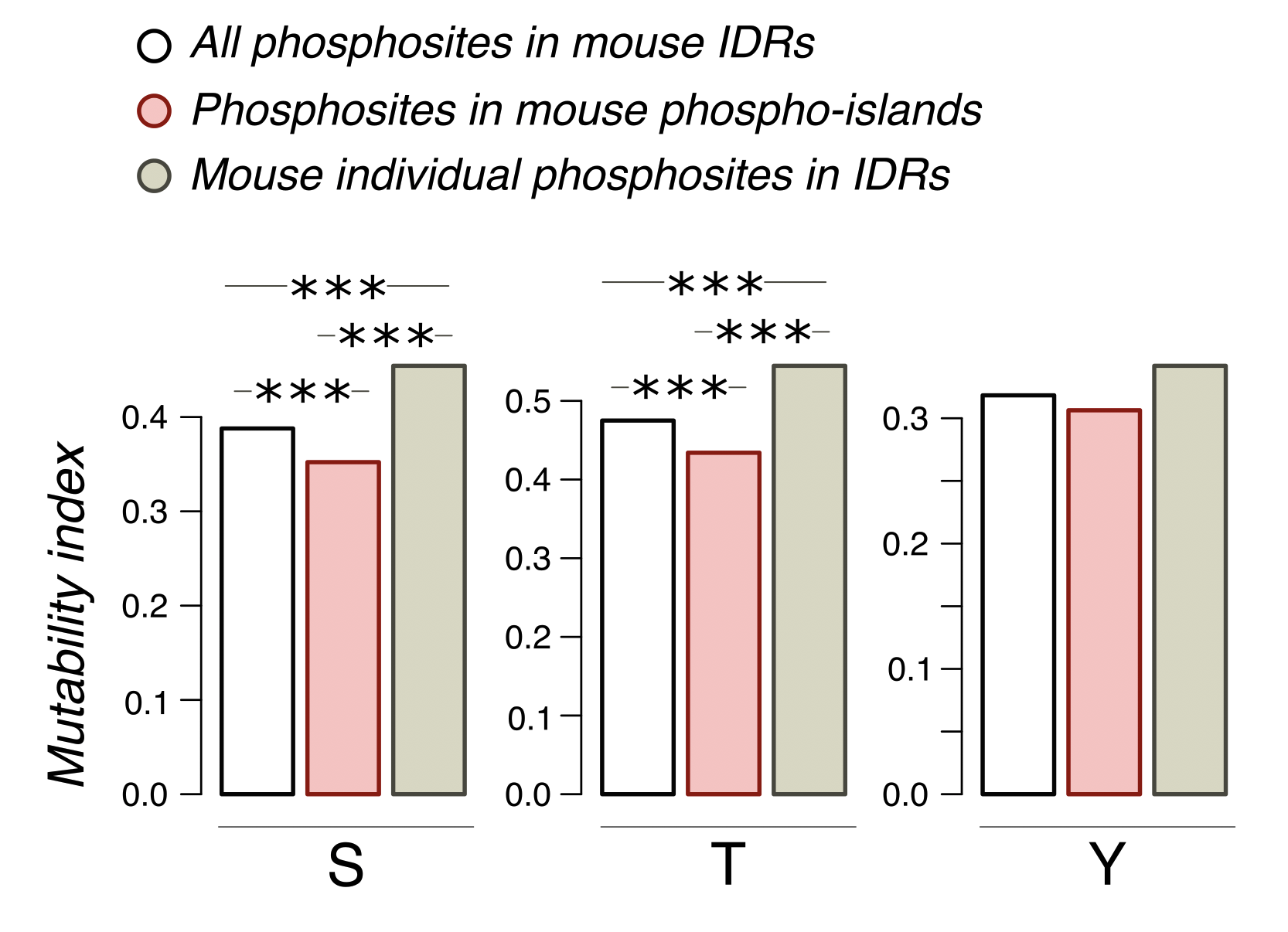


**Supplementary Figure S10 | Conservation of phosphorylated amino acids for various mouse datasets.** The mutability index is calculated for a phosphorylated amino acid with respect to its non-phosphorylated counterpart as the sum of probabilities of STY-to-X mutations calculated for all tree branches for the phosphorylated STY amino acid divided by the same sum for the respective non-phosphorylated amino acid. Three asterisks indicate statistically significant differences (*p* < 0.001, chi-squared contingency test).


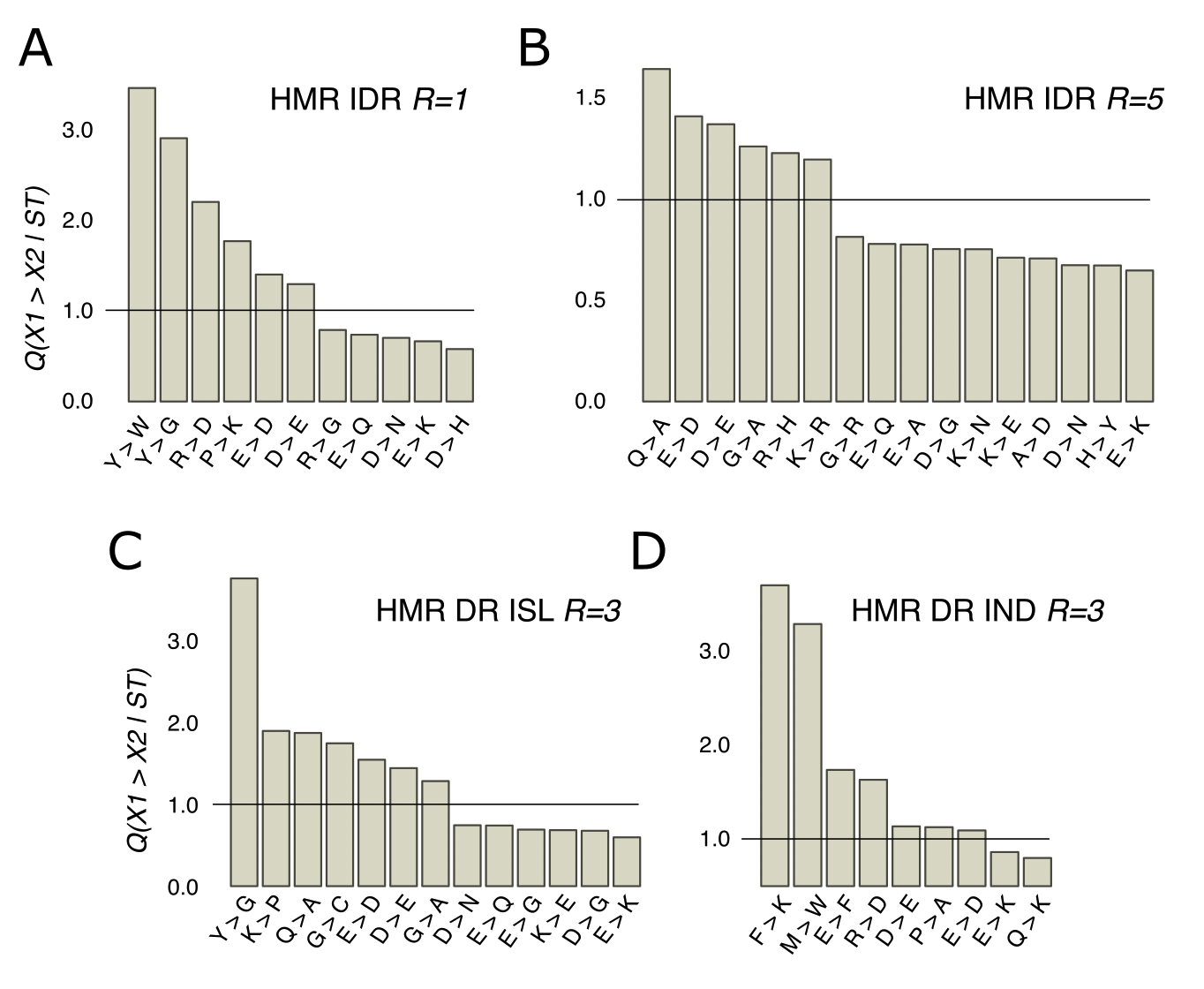

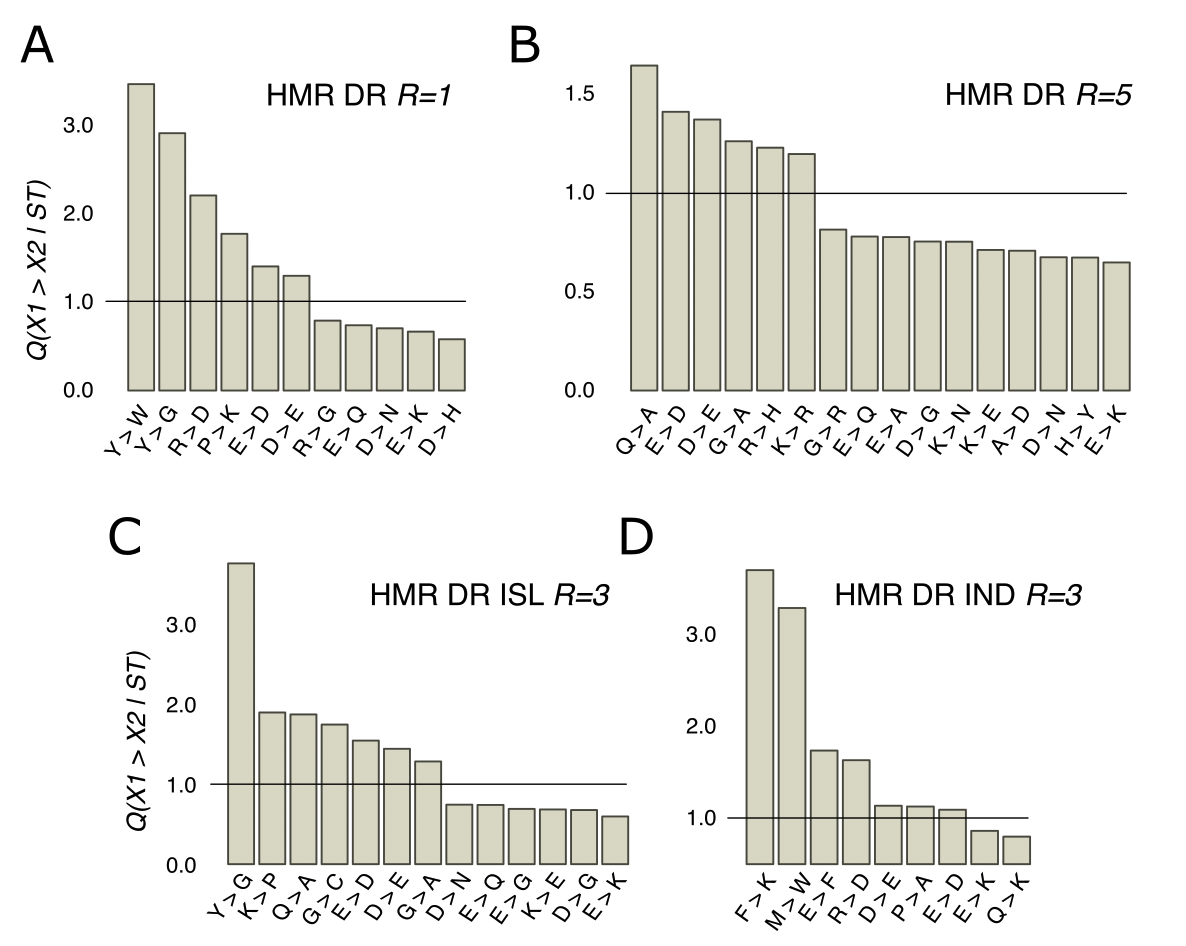


**Supplementary Figure S11 | *R* values of mutations near ST phosphosites with probabilities significantly different from the expected ones for various HMR subsets. (A)** HMR sites located in intrinsically disordered regions with the window radius 1. **(B)** HMR sites located in disordered regions with the window radius 3. **(C)** Clustered HMR sites with the window radius 3. **(D)** Individual HMR sites with the window radius 3.

**2. Supplementary Tables**

Supplementary tables are provided in the additional XLSX file.

**/hmr_sites_list**: List of all HMR-sites considered in the present study. Each site is marked as present in ordered/disordered region, having one of the five motif types, and being a part of a phosphosite cluster or individual. Phosphosites located in IDRs are considered as being neither clustered nor individual.

**/mouse_site_list**: List of mouse dataset phosphosites.

**/hmr_psite_islands**: List of HMR phosphor-islands.

**/mouse_psite_islands**: List of mouse phosphor-islands.

**/R table**: Table with *R* values and the respective corrected *p*-values for all considered phosphosite datasets. Fig. 3 and Suppl. Figs. S2-S6 correspond to this table.

**/local_mutations**: Q values of contextual mutations for different HMR phosphosite datasets and different window radius. Corresponds to Fig. 5 and Suppl. Fig. S9.

**/phosphosite conservation**: Mutability statistics for the mouse dataset. Corresponds to Suppl. Fig. S8.
